# Supplementary material for: Prediction Models for Intrauterine Growth Restriction Using Artificial Intelligence and Machine Learning: A Systematic Review and Meta-Analysis
Source: Healthcare (Basel). 2023 Jun 1;11(11):1617. doi: 10.3390/healthcare11111617 (PMC10252230; doi:10.3390/healthcare11111617)
Supplement: Supplementary file 1 [file healthcare-11-01617-s001.zip › healthcare-2361309-supplementary/Table S1. JBI report.pdf]

Table S1. Summary of quality assessments using JBI appraisal checklist.

Author	Study type	Items on Joanna Briggs Institute										Raw score and %	Risk
		Q1	Q2	Q3	Q4	Q5	Q6	Q7	Q8	Q9	Q10		
Zhiwei Guo	Case Control	1	1	1	1	1	1	1	1	1	1	10/10 = 100%	Low
Sonia Dahdoud	Case Control	1	1	1	1	1	1	1	U	1	1	9/10 = 90%	Low
F Lunghi	Case Control	1	1	1	1	1	0	0	1	1	1	8/10 = 80%	Low
G.Magenes	Case Control	1	1	1	1	1	0	0	1	1	1	8/10 = 80%	Low
Maria G. Signorini	Case Control	1	1	1	1	1	1	1	1	1	1	10/10 = 100%	Low
I.C. Crockart	Case Control	1	1	1	1	1	1	1	U	1	1	9/10 = 90%	Low
Ray Bahado-Singh	Case Control	1	1	1	1	1	1	1	1	1	1	10/10 = 100%	Low
Nicolo Pini	Case Control	1	1	1	1	1	1	1	1	0	1	9/10 = 90%	Low
G. Magenes	Case Control	1	1	1	1	1	1	1	1	1	1	10/10 = 100%	Low
C Xu	Case Control	1	1	U	1	1	1	U	1	1	1	8/10 = 80%	Low
Massimo Buscema	Case Control	1	1	1	1	1	1	0	1	1	1	9/10 = 90%	Low
F. Foltran	Case Control	0	0	0	0	0	1	0	0	U	0	1/10 = 10%	High
Maria E Street	Case Control	1	1	1	1	1	0	0	1	0	1	7/10 = 70%	Low
M. Ferrario	Case Control	1	1	1	1	1	0	0	1	1	U	7/10 = 70%	Low
R Deval	Case-Control	0	0	0	U	1	0	0	0	0	0	1/10 = 10%	High
L Gomes-James	Case-Control	1	0	1	1	1	0	0	1	1	1	7/10 = 70%	Low
N Aslam	Case-Control	1	1	1	1	1	0	0	1	1	1	8/10 = 80%	Low
H Sufriyana	Case-Control	1	1	1	1	1	0	0	1	1	1	8/10 = 80%	Low
F Gürgen	Case-Control	1	1	1	U	U	0	0	1	1	0	5/10 = 50%	Moderate
SN Van	Case-Control	1	1	1	1	1	0	0	1	1	1	8/10 = 80%	Low

JBI Criteria to be scored: Q1. Were the groups comparable other than presence of disease in cases or absence of disease in controls? Q2. Were cases and controls matched appropriately? Q3. Were the same criteria used for identification of cases and controls? Q4. Was exposure measured in a standard, valid and reliable way? Q5. Was exposure measured in the same way for cases and controls? Q6. Were confounding factors identified? Q7. Were strategies to deal with confounding factors stated? Q8. Were outcomes assessed in a standard, valid and reliable way for cases and controls? Q9. Was the exposure period of interest long enough to be meaningful? Q10. Was appropriate statistical analysis used?

1 = Yes, 0 = No, and U = Unclear.

Abbreviations: 1 = Yes; 0 = No; U = Unclear; JBI: Joanna Briggs Institute

Criteria used to rank the risk of bias

i) ≤49% = high risk of Bias (2 Studies)
ii) 50% and 69% = Moderate risk of Bias (1 study)
iii) Above 70% = low risk of Bias (17 studies)
