# Supplementary material for: Prediction Models for Intrauterine Growth Restriction Using Artificial Intelligence and Machine Learning: A Systematic Review and Meta-Analysis
Source: Healthcare (Basel). 2023 Jun 1;11(11):1617. doi: 10.3390/healthcare11111617 (PMC10252230; doi:10.3390/healthcare11111617)
Supplement: Supplementary file 1 [file healthcare-11-01617-s001.zip › healthcare-2361309-supplementary/Table S2. CASP report.pdf]

Table S2. Summary of quality assessments using CASP appraisal checklist.

**Author**	**Study type**	**Items on CASP checklist**											**Raw score and %**	**Risk**
		**Q1**	**Q2**	**Q3**	**Q4**	**Q5**	**Q6**	**Q7**	**Q8**	**Q9**	**Q10**	**Q11**		
Zhiwei Guo	Case Control	1	1	1	0	1	1	1	1	1	1	1	10/11 = 91%	Low
Sonia Dahdoud	Case Control	1	1	1	1	1	1	1	1	1	1	0	10/11 = 91%	Low
F Lunghi	Case Control	1	1	1	0	0	1	1	1	1	1	0	8/11 = 73%	Low
G.Magenes	Case Control	1	1	1	0	1	1	1	1	1	1	1	10/11 = 91%	Low
Maria G. Signorini	Case Control	1	1	1	0	1	1	1	1	1	1	1	10/11 = 91%	Low
I.C. Crockart	Case Control	1	1	1	1	0	1	1	1	1	1	1	10/11 = 91%	Low
Ray Bahado-Singh	Case Control	1	1	1	1	1	1	1	1	1	1	1	11/11 = 100%	Low
Nicolo Pini	Case Control	1	1	1	1	0	1	1	1	1	1	1	10/11 = 91%	Low
G. Magenes	Case Control	1	1	1	0	1	1	1	1	1	1	1	10/11 = 91%	Low
C Xu	Case Control	1	1	1	0	1	1	1	1	1	1	1	10/11 = 91%	Low
Massimo Buscema	Case Control	1	1	1	0	1	1	1	1	1	1	1	10/11 = 91%	Low
F. Foltran	Case Control	1	1	1	0	0	1	1	0	1	1	0	7/11 = 64%	Moderate
Maria E Street	Case Control	1	1	1	0	0	1	1	1	1	1	1	9/11 = 82%	Low
M. Ferrario	Case Control	1	1	1	0	0	1	1	1	1	1	1	9/11 = 82%	Low
R Deval	Case-Control	1	1	0	0	1	0	1	0	1	U	1	6/11 = 54%	Moderate
L Gómez-Jemes	Case-Control	1	1	1	0	1	1	1	1	1	1	1	10/11 = 91%	Low
H Sufriyana	Case-Control	1	1	1	0	1	1	1	1	1	1	1	10/11 = 91%	Low
N Aslam	Case-Control	1	1	1	0	1	1	1	1	1	1	1	10/11 = 91%	Low
F Gürgen	Case-Control	1	1	1	0	1	1	1	1	1	1	1	10/11 = 91%	Low
SN Van	Case-Control	1	1	1	0	1	1	1	1	1	1	1	10/11 = 91%	Low

CASP Criteria to be scored: **Q1**. Is the CPR clearly defined? **Q2**. Did the population from which the rule was derived include anappropriate spectrum of patients? **Q3**. Was the rule validated in a different group of patients? **Q4**. Were the predictor variables and the outcome evaluated in a blinded fashion? **Q5**. Were the predictor variables andthe outcome evaluates in the whole sample selected initially? **Q6**. Are the statistical methods usedto construct and validate the rule clearly described? **Q7**. Can the performance of the rule be calculated? **Q8**. How precise was the estimate of thetreatment effect? **Q9**. Would the prediction rule be reliable and the results interpretable if used for your patient? **Q10**. Is the rule acceptable in your case? **Q11**. Would the results of the rule modify your decision about themanagement of the patient, orthe information you can give to him/her?

1 = Yes, 0 = No, and U = Unclear.

Abbreviations: 1 = Yes; 0 = No; U = Unclear; NA = Not Applicable; CASP; Critical Appraisal Skills Programme.

Criteria used to rank the risk of bias

i) ≤49% = High risk of Bias
ii) 50% and 69% = Moderate risk of Bias (2 Studies)
iii) Above 70% = Low risk of Bias (18 studies)
